# Individualized feedback to change multiple gait deficits in chronic stroke

**DOI:** 10.1186/s12984-019-0635-4

**Published:** 2019-12-23

**Authors:** Kevin A. Day, Kendra M. Cherry-Allen, Amy J. Bastian

**Affiliations:** 1 Center for Movement Studies, Kennedy Krieger Institute, Baltimore, MD USA; 2 Department of Biomedical Engineering, The Johns Hopkins University School of Medicine, Baltimore, MD USA; 3 Department of Physical Medicine and Rehabilitation, The Johns Hopkins University School of Medicine, Baltimore, MD USA; 4 Department of Neuroscience, The Johns Hopkins University School of Medicine, Baltimore, MD USA

**Keywords:** Gait rehabilitation, Stroke, Visual feedback, PCA, Real-time, Biofeedback

## Abstract

**Background:**

Walking deficits in people post-stroke are often multiple and idiosyncratic in nature. Limited patient and therapist resources necessitate prioritization of deficits such that some may be left unaddressed. More efficient delivery of therapy may alleviate this challenge. Here, we look to determine the utility of a novel principal component-based visual feedback system that targets multiple, patient-specific features of gait in people post-stroke.

**Methods:**

Ten individuals with stroke received two sessions of visual feedback to attain a walking goal. This goal consisted of bilateral knee and hip joint angles of a typical ‘healthy’ walking pattern. The feedback system uses principal component analysis (PCA) to algorithmically weight each of the input features so that participants received one stream of performance feedback. In the first session, participants had to explore different patterns to achieve the goal, and in the second session they were informed of the goal walking pattern. Ten healthy, age-matched individuals received the same paradigm, but with a hemiparetic goal (i.e. to produce the pattern of an exemplar stroke participant). This was to distinguish the extent to which performance limitations in stroke were due neurological injury or the PCA based visual feedback itself.

**Results:**

Principal component-based visual feedback can differentially bias multiple features of walking toward a prescribed goal. On average, individuals with stroke typically improved performance via increased paretic knee and hip flexion, and did not perform better with explicit instruction. In contrast, healthy people performed better (i.e. could produce the desired exemplar stroke pattern) in both sessions, and were best with explicit instruction. Importantly, the feedback for stroke participants accommodated a heterogeneous set of walking deficits by individually weighting each feature based on baseline walking.

**Conclusions:**

People with and without stroke are able to use this novel visual feedback to train multiple, specific features of gait. Important for stroke, the PCA feedback allowed for targeting of patient-specific deficits. This feedback is flexible to any feature of walking in any plane of movement, thus providing a potential tool for therapists to simultaneously target multiple aberrant features of gait.

## Background

Gait impairment following stroke often presents with multiple deficits. Some of the most common deficits include decreased paretic leg knee flexion during swing, hip circumduction, step length asymmetry, pelvic tilt, and decreased ankle dorsiflexion [1–5]. Unfortunately, resources (e.g. patient time/finances, therapist time, insurance coverage, etc), are limited, making it difficult to address all existing deficits in a single episode of care. Consequently, therapists are confronted with the challenge of using their clinical judgement to prioritize deficits, serially targeting those that they believe will most improve walking function and independence. Addressing one deficit in isolation of the others may introduce unintended compensations that further impair gait. Indeed, when manipulating a lower-limb movement pattern, lower-limb sagittal plane kinematics (e.g. hip/knee angles) are closely coupled [6–8].Thus, there remains a need for both the systematic prioritization of gait deficits and improvement in the efficiency of training so as to simultaneously address multiple patient-specific deficits.

Real-time visual feedback of gait kinematics has proven useful in altering targeted features of gait in healthy and neurological populations [9–14]. For example, Cherry-Allen et al. used visual feedback of joint angles to increase peak knee angle in people post-stroke [15]. Moreover, visual feedback has been effective in improving gait speed, stride length, and stride width in people post-stroke [16–18]. Still, research protocols using visual feedback of kinematic gait parameters have two prominent issues when looking to improve individual patient deficits: 1) they are focused on altering one feature of walking while leaving others unconstrained and 2) they are predicated on the assumption that the targeted parameter is the most prominent deficit for the entire group of patients included in the particular study. Given the heterogeneity of deficits following stroke, it would be most beneficial to have a system that can accommodate a wide array of walking patterns.

We developed a novel method to generate individualized, yet simple, visual feedback for re-training walking on a treadmill. An innovative element of this process is the use of principal component analysis (PCA) to display a simple ‘summary’ of a multi-dimensional movement pattern that continuously updates on a screen in front of participants as they walk. PCA has applied to motion data in a number of previous studies to characterize whole-body movement in both healthy [19, 20] and pathological populations [21–25]. The question that we ask here is whether this novel, PCA-based visual feedback system can address multiple, patient-specific deficits simultaneously. For stroke patients, we established a goal walking pattern that included four kinematic dimensions (bilateral hip and knee joint angles) of an average ‘healthy’ walking pattern. Each of the four kinematic dimensions was individually weighted based on a participant’s baseline deficits (defined as the difference between baseline walking and the goal walking pattern). Thus, weights varied across participants and were specific to their deficit.

The primary objective of this study was to evaluate the efficacy of our novel visual feedback in altering gait post-stroke. Thus, to contrast performance of participants with chronic stroke who received a control goal walking pattern (i.e. stroke-to-control), we evaluated the performance of healthy, age-matched controls who receive a hemiparetic goal walking pattern (i.e. control-to-stroke) using the same visual feedback. This contrast allows us to further validate our method by investigating the extent to which performance in stroke-to-control was limited by neurological injury compared to limitations imposed by the method itself. We hypothesized that participants in both groups would be able to use this summary visual feedback to simultaneously alter multiple aspects of their gait (albeit to varying extends depending on their impairment) toward the prescribed goal pattern while walking on a treadmill.

## Methods

### Participants

Ten adults with chronic stroke (3 female; age: 59.0 ± 7.4 yr) and ten group age-matched neurologically intact adults (7 female; age: 57.3 ± 6.8 yr) were recruited for this experiment. All participants with chronic stroke met inclusion and exclusion criteria (Table 1). All participants provided written, informed consent before taking part in the experiment. The experimental protocol was approved by the Johns Hopkins Medicine Institutional Review Board.

**Table 1:** Inclusion and Exclusion Criteria

Inclusion criteria	Exclusion criteria
1. Diagnosis of ischemic or hemorrhagic stroke* 2. Ambulatory with or without an assistive device Δ 3. Persistent lower extremity hemiparesis with a score of < 34 on the lower extremity subscale of the Fugl-Meyer 4. Gait speed > 0.40 m/s 5. Paretic leg peak knee flexion < 55 degrees	1. Neurological condition or injury other than stroke 2. Uncontrolled hypertension (> 190/110 mmHg) 3. Cerebellar signs or evidence of cerebellar involvement* 4. Pregnancy Δ 5. Orthopedic or other medical condition that could compromise walking performanceΔ 6. Unable to give informed consent 7. Unilateral spatial neglect (Star Cancellation Test score < 44/54)

Inclusion and exclusion criteria were determined through standard clinical examination procedures as described in the methods, unless otherwise indicated. *information confirmed by a neurologist and/or magnetic resonance imaging (MRI) reading; Δ information determined by self-report

### Clinical assessments

Participants with chronic stroke underwent clinical examination prior to the experiment. To quantify motor impairment we administered the lower extremity subscale of the Fugl-Meyer test (FM-LE) [26]. This test includes 17 items scored on an ordinal scale (0–2) with 34 possible points and higher scores representing less impairment. We measured self-selected and fastest comfortable over ground walking speeds by having participants walk two passes at each speed across a six-meter electronic walkway (Zeno Walkway, ProtoKinetics, Havertown, PA). Baseline knee and hip flexion angles, used to determine study eligibility, were measured using motion capture while participants walked on the treadmill at their self-selected speed. Participants who customarily wore an ankle-foot orthosis continued using these items throughout the study.

We also tested for sensory impairment in participants with chronic stroke. For proprioception testing, participants were supine with their eyes closed. The examiner stabilized the proximal joint segment and passively moved the distal segment to a position above or below the neutral starting position (neutral position was midway through the joint’s range of motion). The participant reported whether the position of the specified joint was above or below the starting position. Paretic hip, knee, and ankle joints were each tested at six different positions (18 total probes). Participants with stroke also completed The Star Cancellation Test, a screening tool that detects the presence of unilateral spatial neglect [27]. Scores less than 44/54 stars cancelled is suggestive of unilateral spatial neglect.

We assessed cognitive function in both participants with chronic stroke and control participants using the Montreal Cognitive Assessment (MoCA) [28]. Scores greater than 26/30 possible points reflect normal cognitive function.

### Motion analysis

**Fig. 1:**
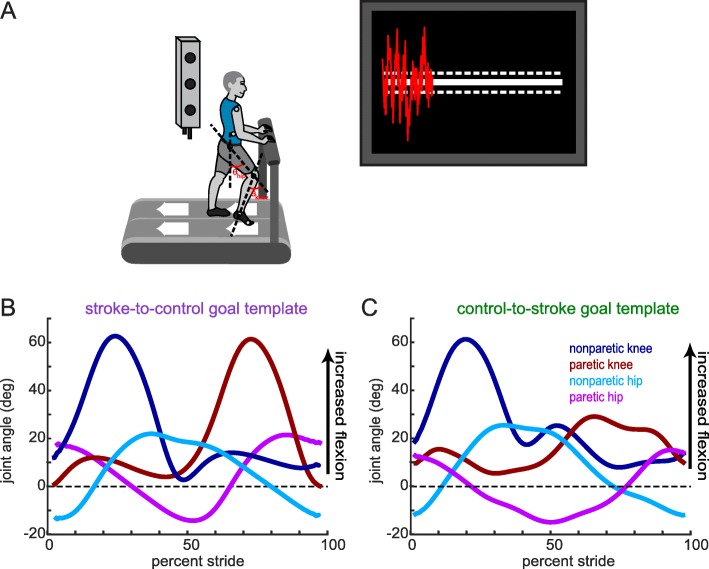
Experimental set-up, visual feedback display and goal walking patterns. (**a**) Marker placement and general set up for motion capture. Bilateral, sagittal-plane hip and knee angles were calculated from the marker positions in real-time. These angles were fed into an algorithm to condense the information into a single dimension. The visual display that participants received is displayed on the right. Participants were instructed to minimize the deviation between their feedback performance (red trace) and the center white target line. Dashed lines around the target line correspond to the success zone. (**b**) Stroke-to-control goal template consisting of the bilateral hip and knee angles of an average control participant while walking. People post-stroke improved their performance using the visual feedback by more closely matching this set of kinematics. (**c**) Control-to-stroke goal template consisting of the bilateral hip and knee angles of an exemplar patient post-stroke with hemiparetic gait affecting the left side. Control participants improved their performance using the visual feedback by more closely matching this set of kinematics

We recorded participants’ kinematics using an Optotrak Certus motion capture system (Northern Digital, Waterloo, ON) as they walked on a split-belt treadmill (Woodway, Waukesha, WI) with a separate belt for each leg. This type of treadmill allowed us to detect right and left foot contacts via distinct force plates under each belt, but the belt speeds were equal throughout all experiments. Kinematic data were collected at 100 Hz from 12 infrared-emitting diodes placed bilaterally on the foot (fifth metatarsal head), ankle (lateral malleolus), knee (lateral epicondyle), hip (greater trochanter), pelvis (iliac crest), and shoulder (acromion process; Fig. 1a).

### Experimental design

**Fig. 2:**
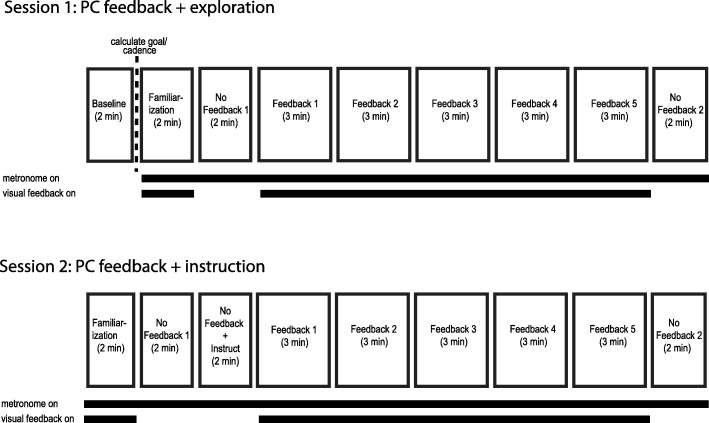
Experimental paradigm. Participants completed two sessions using PC feedback. During Session 1, participants were instructed to use the visual feedback to explore ways in which they could more closely match the goal kinematics. During Session 2, participants were explicitly instructed of how they must alter their walking pattern in order to more closely match the goal kinematics. Participants received 5 three-minute blocks of visual feedback in each session

The study included 2 groups of participants (stroke-to-control and control-to-stroke). Stroke-to-control consisted of participants with stroke who received a goal walking pattern based on a typical ‘healthy’ gait (Fig. 1b, left). Control-to-stroke, on the other hand, consisted of neurologically intact participants who received a goal walking pattern based on a typical post-stroke, impaired gait pattern (Fig. 1b, right). Both groups participated in two sessions at least 3 days apart (median time between session: 27 days for stroke-to-control, 28 days for control-to-stroke) and received the same type of visual feedback (explained below in *Visual Feedback* subsection). During Session 1 (visual feedback + exploration, Fig. 2), participants did not receive information about the goal walking pattern and were instructed to use the visual feedback to explore ways in which to improve their performance. During Session 2 (visual feedback + instruction, Fig. 2), participants were given explicit instructions about how to change their baseline walking in order to progress toward the goal walking pattern and improve their performance using the visual feedback. Session 2 was intended to investigate 1) if participants’ Session 1 performance was limited by incomplete exploration and 2) if the visual feedback offered additional improvement over verbal instruction.

### Goal walking pattern calculation

Participants in the stroke-to-control group received a ‘control’ goal walking pattern while participants in the control-to-stroke group received a stroke goal walking pattern. These goal walking patterns consisted of the sagittal plane knee and hip angles for both legs. We defined knee angle as the angle between the vector connecting the hip and knee marker and the vector connecting the knee and ankle marker (Fig. 1a). We defined hip angle as the angle between the vector connecting the hip and knee marker and a vertical vector (Fig. 1a). The control goal walking pattern was obtained by averaging the baseline walking of 10 neurologically intact individuals who did not take part in this study (Fig. 1b). This control goal walking pattern was collected at 1 m/s. The stroke goal walking pattern was obtained by taking the baseline walking of an exemplar participant with stroke who displayed decreased paretic leg knee and hip flexion during swing (Fig. 1c). This participant was walking at his self-selected speed (0.885 m/s).

**Fig. 3:**
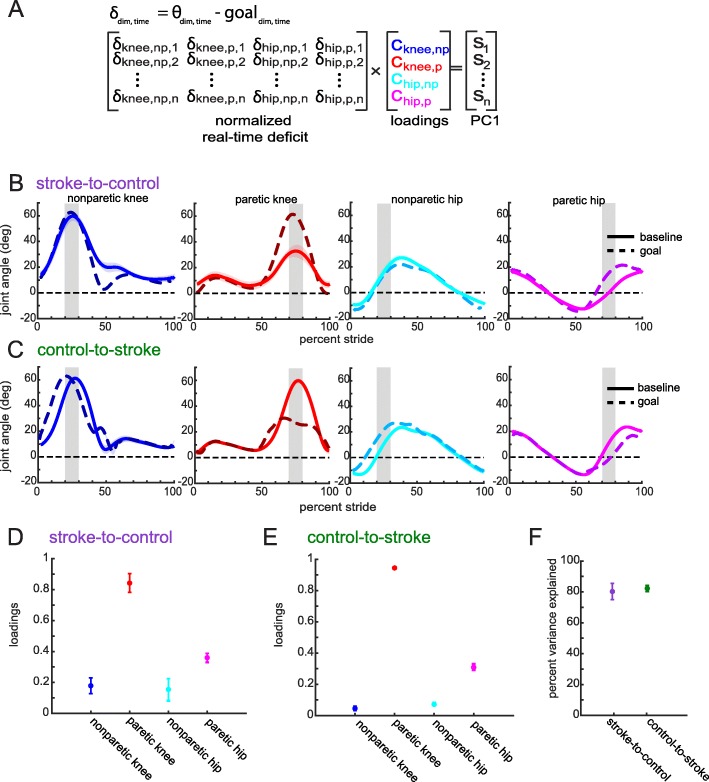
Inputs for the calculation of real-time principal component for visual feedback display. (**a**) Loadings for each dimension were calculated by submitting each individual’s baseline deficit to principal component analysis (PCA). These loadings were used to calculate a real-time principal component (PC1) which is a vector multiplication of the normalized real-time deficit (defined as the difference between the z-scored real-time kinematics and the prescribed goal kinematics) and the loadings. (**b**) Stroke-to-control group average baseline kinematics (solid traces) plotted against goal kinematics (dashed traces) for the four input dimensions. The shaded gray regions denote the rewarded time window during swing phase for that particular dimension (**c**) Control-to-stroke group average baseline kinematics (solid traces) plotted against goal kinematics (dashed traces) for the four input dimensions. The shaded gray regions denote the rewarded time window during swing phase for that particular dimension. The 10 % width of these windows is only approximate as the time window was fixed at 100 ms for each individual (**d**) Stroke-to-control group average loadings for the four input dimensions. (**e**) Control-to-stroke group average loadings for the four input dimensions. (**f**) Percent variance explained by the first principal component. All shaded errors and error bars denote SEM

We chose to focus on altering kinematics during the swing phase on each leg because kinematics during stance are largely constrained by the speed of the treadmill belts. Therefore, we set the goal during stance for each individual to the kinematics observed during their baseline walking. To obtain the individualized goal during swing, we resampled the goal templates over the stride time of each particular individual (Fig. 3b, c).

### Visual feedback

Participants received PC visual feedback during each of their two sessions. This feedback consisted of a principal component-based visual feedback in which information from the four kinematic dimensions (bilateral knee and hip angles) was condensed to a one-dimensional summary of performance.

Bilateral lower limb marker positions were sampled from the Optotrak software and fed into a custom Python program in real-time. From these positions, we calculated sagittal plane hip and knee angles over the stride cycle. Visual feedback was displayed using a Vizard development environment (WorldViz, Santa Barbara, CA) and reflected the participants’ step-by-step deviation from the desired pattern. This real-time information was presented as a red trace on the screen overlaid on a white goal zone that centered on a white target line (defined as ±0.25 a.u., Fig. 1a). This outer-bound was selected based on the variability observed during baseline walking in pilot testing. Subjects were instructed to change their walking pattern so that the red feedback line stayed within the goal zone and as close to the target line as much as possible (e.g. minimal deviation from the goal). If a participant matched the goal pattern exactly (i.e. difference of zero), the red feedback trace would overlap the white target line. The visual feedback was updated upon each heel strike (i.e. two new data points per stride) and tracked across the TV screen so that participants had information of their current and past performance.

The principal component-based feedback weighted each of the input kinematic dimensions based on each individual’s deficits. These weights (also termed loadings) were calculated from baseline walking by submitting the difference between baseline and goal kinematics (i.e. the deficit) to the PCA (Fig. 3a). Thus, real-time performance in PC space could be calculated by a vector multiplication of the real-time deficit (i.e. difference between current kinematics and goal) and the loadings (Fig. 3a). Loadings were kept constant across all sessions so that the participants had a constant mapping between a change in their kinematics and their feedback performance.

While using the visual feedback, locking stride time was necessary as we had to set a length of time for the goal stride from which to compare real-time performance. Thus, we used a metronome to standardize the participants’ walking stride time. Participants were instructed to heel-strike in rhythm with the beat from the metronome. The metronome pacing was calculated from each individual’s average stride time during baseline walking.

Feedback was calculated at approximately halfway through the swing phase of each leg and displayed at heel strike. Thus, feedback given at right leg heel strike contained information from the preceding right leg swing and left leg stance phase, and vice versa for the feedback given at left leg heel strike. This delay corresponds to approximately ¼ of the stride cycle (i.e. 300–350 ms on average).

### Experimental paradigm

Participants walked on a custom-built treadmill which was also controlled through Vizard. For stroke-to-control, walking speed was set to participants’ self-selected walking speed for all walking trials (the belts were always tied at the same speed). For control-to-stroke, walking speed was set to the selected speed in which the exemplar stroke kinematics were collected (0.885 m/s). Prior to testing, we confirmed that the 0.885 m/s belt speed was a comfortable pace for all participants in the control-to-stroke group. Participants were instructed to stand in the middle of the treadmill with one foot on each belt so that we could detect heel strikes from the force plates for visual feedback display. They wore a safety harness that was suspended from the ceiling to protect against the risk of falling. The harness did not provide any body weight support. All participants in both groups were instructed to hold on to a handrail in front of them (Fig. 1a).

Session 1 (PC feedback + exploration) consisted of 9 walking blocks (Fig. 2). *Baseline*: 2 min of walking naturally. From this block, we calculated the goal walking pattern and goal stride time. *Familiarization:* 2 min of walking with the visual feedback and metronome. The goal walking pattern was set to each individual’s baseline kinematics and participants were informed that performance during this block did not matter and they were free to explore using the feedback. *No Feedback 1:* 2 min of walking naturally to the beat of the metronome. Participants did not receive performance feedback. This block served as our measure of baseline performance. *Feedback 1–5:* 5 identical, 3 min blocks of walking with visual feedback to the beat of the metronome. Participants were instructed to change their walking pattern to get as close to the target line as possible. Importantly, participants were not informed of the goal walking pattern and were instructed to use the visual feedback to determine which walking patterns improved their performance. Participants were free to take rests (either sitting or standing) in between blocks. *No Feedback 2:* 2 min of walking without the visual feedback but still in rhythm with the metronome. Participants were instructed to replicate the walking pattern that maximized their performance during the preceding feedback blocks. This block served as our measure of retention of performance.

Session 2 (PC feedback + instruction) was similar to Session 1 with the exception of an included *No Feedback + Instruct* block following *No Feedback 1* (Fig. 2). During this block, participants were explicitly told how to change their walking to more closely match the goal kinematics. For example, control-to-stroke participants were told to stiffen their left leg (i.e. keep their hip and knee more extended during swing) while walking naturally with their right leg. Instructions for stroke-to-control participants depended on the individual deficit and were directional in nature (i.e. more, less, faster, slower, etc). For example, a participant with deficits in paretic leg flexion and no deficits on the nonparetic leg would be instructed to keep their nonparetic leg constant while bending their paretic knee more and bringing their paretic hip through faster during swing. The *No Feedback + Instruct* block was intended to measure the influence of solely verbal instruction on participants’ performance. Thus, participants did not receive visual feedback during this block. For *Feedback 1–5,* participants were reminded of the instruction prior to each of these feedback blocks and were instructed to use the visual feedback to hone in on the exact goal walking pattern we were instructing. Session 2 was designed to observe whether incomplete exploration had limited performance during Session 1.

### Data analysis

Our primary measure of performance is the mean difference from goal in PC space (measured in arbitrary units) over the course of training. This measure gives a standardized step-by-step difference between the current performance and the prescribed goal pattern, and is calculated as a linear transformation from the real-time kinematics using the individualized loadings (Fig. 3a). These differences are calculated and averaged over the rewarded time window (100 millisecond windows centered approximately around mid-swing of each leg; displayed as the shaded gray regions between 20 and 30 and 70–80% stride in Fig. 3b, c). A value of zero represents perfect performance in which the current kinematics exactly match the goal walking pattern within the rewarded time windows. We calculated the difference between *No Feedback 1* and *Feedback 5* as our measure of training and the difference between *Feedback 5* and *No Feedback 2* as our measure of retention of performance. Two participants in stroke-to-control did not experience a *No Feedback 2* block so our retention measure for Session 1 was calculated for the remaining eight participants. We were able to collect all blocks for all participants in control-to-stroke. For Session 2, we calculated the difference between *No Feedback 1* and *No Feedback + Instruct* to measure the effect of verbal instruction on performance. We also included a measure of training beyond instruction (difference between *No Feedback + Instruct* and *Feedback 5*) and a measure of retention (difference between *Feedback 5* and *No Feedback 2*). Due to travel restrictions, one participant from stroke-to-control was not able to return for Session 2. All participants in control-to-stroke completed both sessions.

In addition, we measured specific kinematic features over the course of training. Specifically, we observed the hip and knee flexion angles within the rewarded time window. For clarity in reporting, we refer to the control participants’ left leg as the ‘paretic’ leg and right leg as the ‘nonparetic’ leg as these terms correspond to the goal set of kinematics they are trying to match for the exemplar stroke walking pattern. We were also interested if training increased the amount of ankle clearance present in the stroke-to-control group. As such, we calculated the average step-by-step vertical dimension of the ankle marker during late swing of the paretic foot (defined as mid-swing to heelstrike).

To better understand how participants changed their kinematics during training, we conducted an analysis to calculate the percentage of steps taken outside of their natural walking variability. We defined this measure as *percent improvement*. To define each participant’s natural walking variability, we calculated the paretic hip and knee flexion during mid-swing of each step within No *Feedback 1* and fit a 95% confidence ellipse to those joint angles. For subsequent training blocks, we calculated the percentage of steps that were both outside of this baseline confidence ellipse and resulted in improved performance (i.e. closer to the PC goal). This allowed us to obtain a single measure that quantified a participant’s change in kinematics beyond their baseline walking for a given feedback block.

Participants were instructed to walk in rhythm with the metronome. We calculated stride time as the time between successive heel-strikes (measured in milliseconds).

### Statistical analysis

To identify differences between group demographics (e.g. age, MoCA score, etc.) we used independent samples t-tests. When comparing loading values within and across groups, we used mixed-design analysis of variance (ANOVA) with *dimension* and *group* main factors. A linear least squares regression was used to relate loading values to the deficit for individual kinematic dimensions in stroke-to-control. To confirm that participants remained within 60 ms (approx. 5%) of the prescribed stride time, we performed right-tailed, one-sample t-tests on the absolute difference between participants’ stride times during all blocks of the experiment and the metronome beat interval against a null hypothesis of mean 60. As detailed in ‘Data Analysis’, we were interested in specific a priori performance measures in PC space across blocks (i.e. training, retention for Session 1; instruction, training beyond instruction, and retention for Session 2).

When comparing each kinematic dimension across training in Session 1 (i.e. *No Feedback 1* to *Feedback 5)*, we used paired sample t-tests. When performing this analysis for Session 2 (with *No Feedback 1, No Feedback + instruct,* and *Feedback 5* blocks included), we used repeated-measures ANOVA with a main factor of *block*. To compare changes in joint angles across groups, we used mixed-design ANOVA with *joint* and *block* as within-subject factors and *group* as a between-subject factor. To observe how these joint angle changes affected the end effecter (i.e. ankle) over training, we used a linear least squares regression on the ankle clearance during late swing over trials of training.

To compare *percentage improvement* across blocks and groups, we used mixed-design ANOVA main factors with *block* and *group*. We performed a separate ANOVA for each session. For all repeated-measures ANOVA, we performed Mauchly’s test of sphericity and used the Greenhouse-Geisser correction if degrees of freedom if sphericity was violated. Post-hoc analysis was performed using the Bonferonni correction for multiple comparisons. All outcome measures were tested for normality with the Shapiro-Wilk test and submitted to nonparametric analyses if non-normal. When using mixed-design ANOVA on non-normal data, we used ranked data. All nonparametric tests and test statistics are reported in text. All analyses were performed using SPSS 25.0 (IBM, Armonk, NY) and α–level was set to 0.05.

## Results

Table 2 summarizes the demographic data for the stroke-to-control group. Participants in the control-to-stroke group were recruited so as to be group average age-matched to participants within the stroke-to-control group (t_18_ = − 0.53, *p* = 0.600). Average MoCA scores in stroke-to-control were lower than those observed in our age-matched controls (stroke-to-control: 23 ± 2.9; control-to-stroke were 28.1 ± 1.0; t_18_ = 2.14, *p* = 0.047).

**Table 2:** Demographic Data

	1	2	3	4	5	6	7	8	9	10	Group
Age (years)	57	51	48	68	61	53	62	55	65	70	59.0 ± 7.4
Gender (male/female)	M	F	M	F	M	F	M	M	M	M	7/3
Time post stroke (months)	78	35	84	101	75	11	94	29	93	57	65.7 ± 31.1
Dominant side (right/left)	R	R	R	R	R	R	R	R	R	R	10/0
Paretic side (right/left)	L	L	L	R	L	L	R	L	L	L	2/8
Self-selected gait speed (m/s)	0.885	0.739	1.175	0.736	0.514	0.510	0.741	0.426	0.641	0.700	0.707 ± 0.214
Baseline paretic knee flexion (degrees)	41 1	33.5	49.3	34.4	35.4	48.9	36.5	9.3	28.4	31.4	34.8 ± 11.3
Baseline paretic hip flexion (degrees)	22.0	20.5	20.1	19.5	23.8	15.6	16.2	9.9	10.9	16.9	17.5 ± 4.6
LE-FM score	25	26	25	26	21	22	31	29	30	24	25.9 ± 3.3
Proprioception (intact/impaired)	+	–	+	+	+	+	+	–	+	+	8/2
MoCA score	26	26	30	30	21	27	27	23	23	27	26.0 ± 2.9
Return for session 2 (y/n)	y	y	y	y	y	y	y	n	y	y	9/1

Demographic data for individual subjects. Group data are means ± SD or counts. M: male, F: female; R: right, L: left; P: paretic, NP: non-paretic; LE-FM: Lower extremity subscale for the Fugl-Meyer; out of 34, higher is less impairment. Proprioception: out of 18 total responses (6 probes at 3 joints); +: intact indicates that all responses were correct, −: impaired indicates at least one response was incorrect. MoCA: Montreal Cognitive Assessment; out of 30, higher is less impairment

The goal of this experiment was to evaluate the use of principal component-based visual feedback to bias participants’ walking toward the prescribed goal. The feedback contained four input kinematic dimensions (bilateral hip and knee joint angles, Fig. 3a). Notably, participants in both groups experienced the largest deficits (defined as the difference between baseline and goal walking) in paretic knee and hip flexion (Fig. 3b, c). On average, stroke-to-control needed to increase the amount of flexion during the rewarded time windows to approach the goal (Fig. 3b) while control-to-stroke needed to decrease the amount of flexion observed during the rewarded time window (Fig. 3c).

### Patient-specific weighting of input dimensions

These general directional goals for each group are reflected in the loading values (Fig. 3d, e). The loadings reflect the amount that each dimension is weighted when calculating the principal component (Fig. 3a). A loading value of 1 corresponds to a highly-weighted dimension while a value of 0 corresponds to a dimension that is unweighted. Both the stroke-to-control and control-to-stroke groups are most heavily loaded on the paretic knee and paretic hip. Nonparetic knee and nonparetic hip are less heavily loaded for both groups. Specifically, a mixed-design ANOVA with *dimension* and *group* main factors reveals a significant *dimension* effect (F_1.31, 23.56_ = 183.27, *p* < 0.001) in which post-hoc analysis shows the paretic knee is more heavily loaded than the nonparetic knee (p < 0.001) and the paretic hip is more heavily loaded than the nonparetic hip (p < 0.001). Additionally, the paretic knee is more heavily loaded than the paretic hip (p < 0.001). Dimensions were similar across groups, as evidenced by a nonsignificant *group* effect (F_1,18_ = 2.37, *p* = 0.141).

**Fig. 4:**
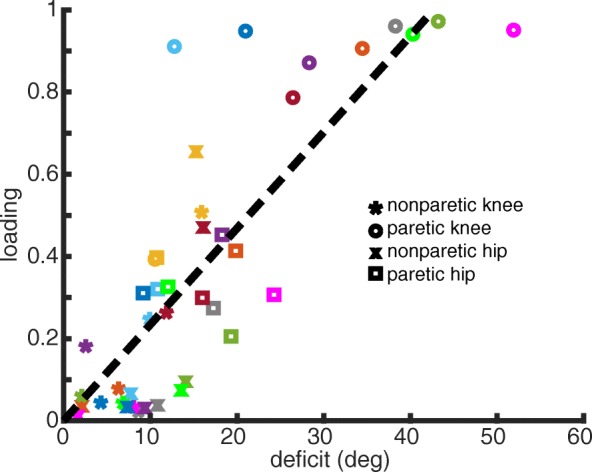
Individualization of visual feedback based on baseline walking deficits in stroke. Each participant is denoted by a separate color with different symbols corresponding to one of the four input dimensions. The dashed line corresponds to a best-fit linear relationship determined by least-squares linear regression. This line can be described by y = 0.023*x + 0.0003 (R^2^ = 0.685, *p* < 0.001). Note, the larger the deficit in a given dimension, the more heavily weighted it is when calculating the PC feedback

Determining the loadings based on the deficit for each input dimension allows for individualization of the visual feedback based on patient-specific deficits. Figure 4 displays the positive relationship (R^2^ = 0.684, p < 0.001) between the magnitude of a participant’s deficit in a given dimension and the corresponding PC loading. Thus, participants in stroke-to-control who display varying levels of deficits in their baseline walking will receive loadings on the dimensions in which there exists greater discrepancy between baseline walking and the goal pattern.

### Session 1 performance

**Fig. 5:**
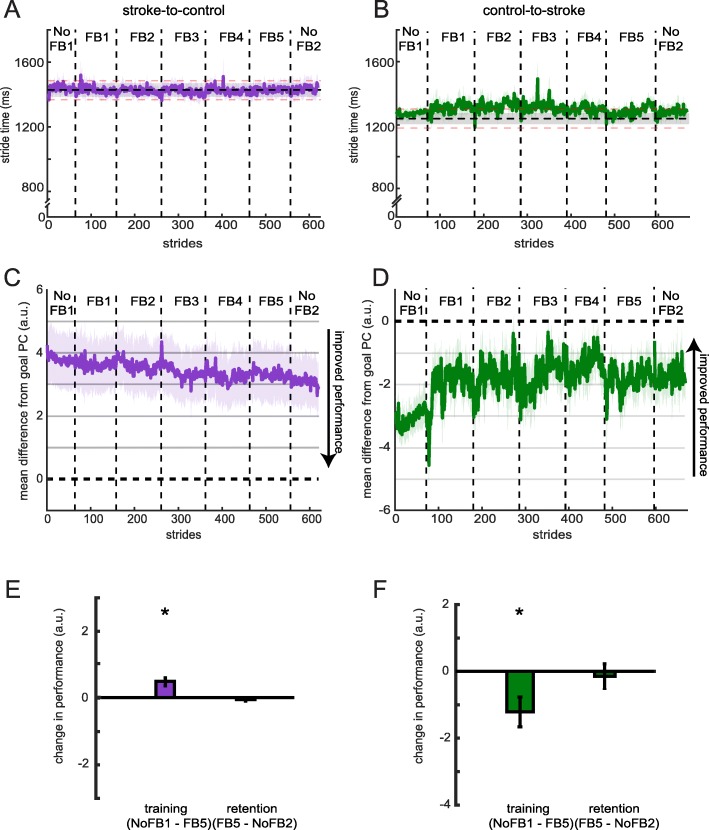
Performance during Session 1. Stride times across blocks for (**a**) stroke-to-control and (**b**) control-to-stroke groups. The horizontal shaded dashed line corresponds to the group average ± SEM stride time provided by the metronome. Red dashed lines correspond to ±60 ms (approx. 5%) from the mean step time provided by the metronome. Group performance in PC space for (**c**) stroke-to-control and (**d**) control-to-stroke groups. . Differences were calculated during the rewarded time windows for each step (i.e. mid-swing). Perfect performance is reflected by a value of 0. Measures of performance change are displayed for (**e**) stroke-to-control and (**f**) control-to-stroke groups. * denotes a significant change between blocks (*p* < 0.05). All error bars denote SEM

Figure 5a and b display the stride times during all blocks of Session 1 for stroke-to-control and control-to-stroke, respectively. Participants in both groups were able to stay within 60 ms (approx. 5%; red dashed lines, Fig. 5a, b) of this prescribed walking cadence during all blocks (all *p* > 0.937 for stroke-to-control, Fig. 5a; all *p* > 0.065 for control-to-stroke, Fig. 5b).

Our primary measure of performance was the stride-by-stride difference from the goal principal component (measured in a.u., Fig. 5c, d). This measure is a composite of all weighted input dimensions. Thus, values closer to zero correspond to improved performance in this task. We were first interested in the effect of the feedback on performance (i.e. training) and used the difference in performance between *No Feedback 1* and *Feedback 5* to quantify this effect. Both stroke-to-control (t_9_ = 4.16, *p* = 0.003; Fig. 5e) and control-to-stroke (t_9_ = − 2.67, *p* = 0.026; Fig. 5f) showed significant improvement towards the performance goal due to training with the visual feedback. When comparing the absolute improvement across groups, we observed that the control-to-stroke group demonstrated a larger improvement in this measure than stroke-to-control (t_18_ = 2.94, *p* = 0.009). Both groups displayed similar absolute levels of performance at baseline (i.e. *No Feedback 1*; t_18_ = 0.77, *p* = 0.451).

We were also interested if participants in both groups were able to retain their level of performance when the visual feedback was turned off. During *No Feedback 2*, participants were instructed to replicate the walking pattern that allowed them to achieve their best performance while using the visual feedback. We used the difference in performance between *Feedback 5* and *No Feedback 2* to quantify this effect. Performance did not significantly differ between blocks for either stroke-to-control (t_7_ = − 1.40, *p* = 0.203; Fig. 5e) or control-to-stroke (t_9_ = − 0.40, *p* = 0.700; Fig. 5f), despite the removal of visual feedback.

**Fig. 6:**
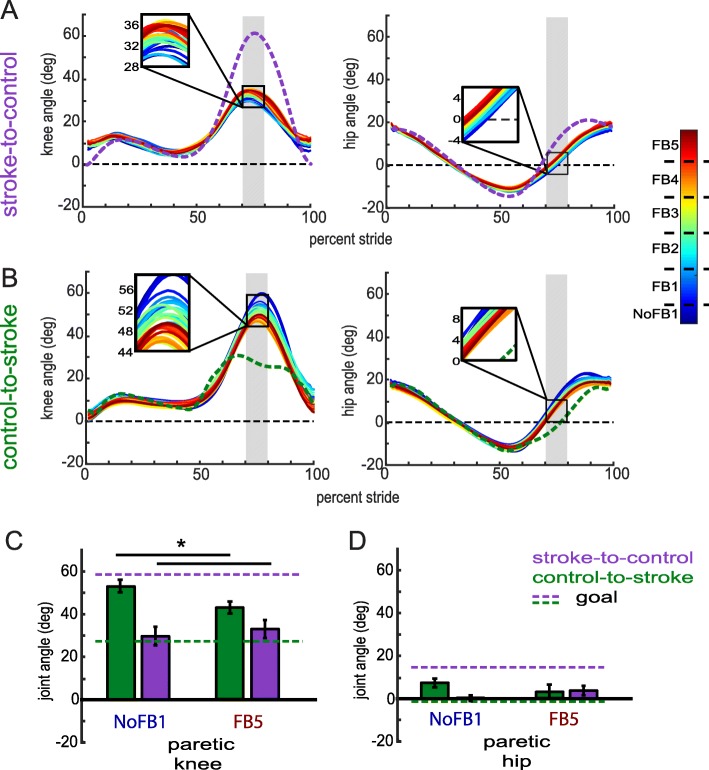
Paretic leg kinematics during Session 1. (**a**) Paretic knee (left) and hip (right) kinematics for stroke-to-control group. (**b**) Paretic knee (left) and hip (right) kinematics for control-to-stroke group. Cold colors denote early training while warm colors denote late training. The shaded gray region denotes the rewarded time window during mid-swing. Insets display magnified kinematics during this time window. Colored dashed lines correspond to the goal kinematics for that given dimension. (**c**) Group average paretic knee flexion angles within the rewarded time window during No Feedback 1 and Feedback 5 blocks. (**d**) Group average paretic hip flexion angles within the rewarded time window during No Feedback 1 and Feedback 5 blocks. Colored dashed lines correspond to the goal for that given dimension (purple for stroke-to-control, green for control-to-stroke). * denotes a between-subject difference (*p* < 0.05) and all error bars denote SEM

To observe which walking features participants were modifying during training, we analyzed each kinematic dimension separately. Figure 6 displays the kinematics over training for the paretic knee and paretic hip for both groups. To improve in the task, participants in the stroke-to-control group were required to increase flexion during swing in these dimensions while participants in the control-to-stroke group were required to decrease flexion during swing in these dimensions. We observed that participants were indeed able to adjust their kinematics toward the goal walking pattern over the course of training (Fig. 6a, b). When comparing mean joint angles between *No Feedback 1* and *Feedback 5*, we observed that participants in the stroke-to-control group trended toward increasing flexion in their paretic hip (t_9_ = − 2.08, *p* = 0.067; Fig. 6d) and showed a robust increase in flexion in their paretic knee (Wilcoxon signed rank test Z = 2.70, *p* = 0.007; Fig. 6c). Participants in control-to-stroke demonstrated decreased flexion in the paretic knee (t_9_ = 2.96, *p* = 0.016; Fig. 6c) but not the paretic hip (t_9_ = 1.21, *p* = 0.258; Fig. 6d). Overall, we were able to demonstrate that PC feedback was successful in driving kinematics toward the respective goal for each group during a single session. That is, stroke-to-control tended to increase flexion in paretic hip and knee angles while control-to-stroke tended to decrease flexion in paretic hip and knee angles. Specifically, mixed-design ANOVA of ranked data with *block* and *joint* within-subject factors and a *group* between-subject factor revealed a *block x group* interaction (F_1,18_ = 6.84, *p* = 0.018). Post-hoc analysis revealed that this interaction was driven by a significant difference between groups during *No Feedback 1* (mean pairwise difference = 16.6 degrees; *p* = 0.001), which was reduced by *Feedback* 5 (mean pairwise difference = 3.9 degrees; *p* = 0.452). Thus, participants in each group displayed directional bias in their kinematics toward their respective goal while using the feedback, such that group kinematics more closely matched each other after training.

**Fig. 7:**
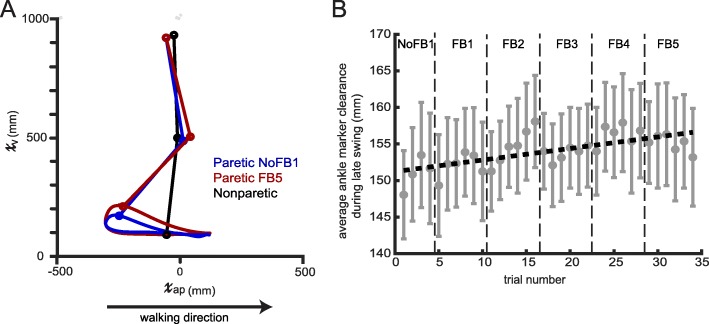
Ankle clearance over training during Session 1. (**a**) Sagittal plane paretic-leg ankle trajectory of example stroke patient prior to training (No Feedback 1, blue) and during late training (Feedback 5, red). Nonparetic leg during mid-swing of the paretic leg is drawn in black. Notice the increased clearance during mid-swing as well as the cleaner heelstrike during late training (**b**) Average ankle marker clearance (defined as the mean vertical position of the ankle from mid-swing to heelstrike) over training. The dashed black line is a linear fit calculated by a least-squares linear regression. This line can be described by y = 0.158*x + 151.223 (R^2^ = 0.447, *p* < 0.001). Each trial on the x-axis corresponds to 30 s of walking (approx. 25 strides). Error bars denote SEM

Many people with post-stroke paretic gait have decreased foot clearance during swing of their affected leg, which increases the risk of tripping [29–32]. We found that the combination of increased paretic hip and knee flexion achieved through a single session of training with this protocol can also lead to increased vertical ankle clearance (Fig. 7a shows example participant). We defined ankle marker clearance as the average vertical distance from the treadmill belt during late swing (i.e. mid-swing to heel-strike). We chose ankle clearance instead of toe clearance because our visual feedback did not directly target ankle plantar/dorsiflexion; thus, the ankle is the end effecter for this particular training. Figure 7b shows that training using the PC visual feedback causes a gradual linear increase (R^2^ = 0.447, *p* < 0.001) in the vertical clearance of their ankle during late swing (0.158 mm/trial; 1 trial is 30 s of walking containing approx. 25 strides).

### Session 2 performance

**Fig. 8:**
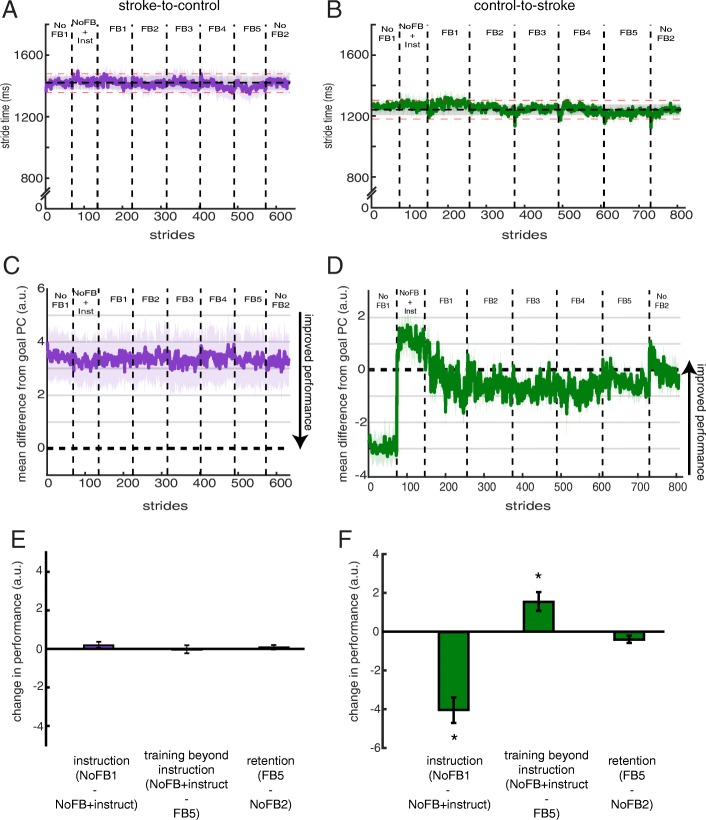
Performance during Session 2. Stride times across blocks for (**a**) stroke-to-control and (**b**) control-to-stroke groups. The horizontal shaded dashed line corresponds to the group average ± SEM stride time provided by the metronome. Red dashed lines correspond to ±60 ms (approx. 5%) from the mean step time provided by the metronome. Group performance in PC space for (**c**) stroke-to-control and (**d**) control-to-stroke groups. . Differences were calculated during the rewarded time windows for each step (i.e. mid-swing). Perfect performance is reflected by a value of 0. Measures of performance change are displayed for (**e**) stroke-to-control and (**f**) control-to-stroke groups. * denotes a significant change between blocks (*p* < 0.05). All error bars denote SEM

Figures 8a and b display the stride times during all blocks of Session 2 for stroke-to-control and control-to-stroke, respectively. Similar to Session 1, participants in both groups were able to stay within 60 ms (approx. 5%; red dashed lines, Fig. 8a, b) of this prescribed walking cadence during all blocks (all *p* > 0.316 for stroke-to-control, Fig. 8a; all *p* > 0.764 for control-to-stroke, Fig. 8b).

During Session 2, we wanted to remove the potential performance limitations that could result from insufficient movement exploration. As such, we provided explicit instructions about the goal walking pattern and details about how to alter baseline walking to improve performance using the feedback. Figure 8c and d display performance in PC space during Session 2 for stroke-to-control and control-to-stroke, respectively. As a measure of effect of verbal instruction on performance, we calculated the difference in PC space between *No Feedback 1* and *No Feedback + Instruct* blocks. Stroke-to-control was not able to improve their performance over baseline (t_8_ = 1.27, *p* = 0.241, Fig. 8e) while control-to-stroke experienced a large change in performance (t_9_ = − 6.14, *p* < 0.001, Fig. 8f) and actually overshot the target, given the instruction. When given visual feedback to improve performance beyond the level observed due to solely verbal instruction, the stoke-to-control group was again unable to alter performance (Wilcoxon signed ranks test Z = -0.30, *p* = 0.767, Fig. 8e) while the control-to-stroke group was able to hone in on the goal pattern and use the visual feedback to improve their performance beyond instruction (t_9_ = 3.24, *p* = 0.010, Fig. 8f). Once the visual feedback was turned off in *No Feedback 2* and participants were instructed to replicate the walking pattern that produced the best performance, participants in the control-to-stroke group were able to maintain the performance observed during *Feedback 5* (t_9_ = − 2.15, *p* = 0.06, Fig. 8f)*;*albeit performance actually trended toward the goal target (Fig. 8d).

**Fig. 9:**
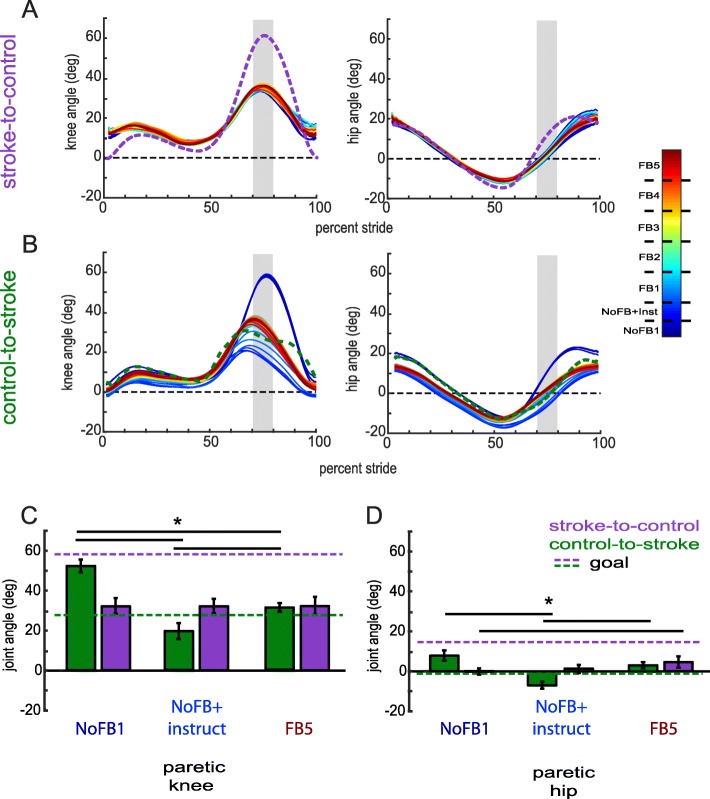
Paretic leg kinematics during Session 2. (**a**) Paretic knee (left) and hip (right) kinematics for stroke-to-control group. (**b**) Paretic knee (left) and hip (right) kinematics for control-to-stroke group. Cold colors denote early training while warm colors denote late training. The shaded gray region denotes the rewarded time window during mid-swing. Colored dashed lines correspond to the goal kinematics for that given dimension. (**c**) Group average paretic knee flexion angles within the rewarded time window during No Feedback 1, No Feedback + Instruct, and Feedback 5 blocks. (**d**) Group average paretic hip flexion angles within the rewarded time window during No Feedback 1, No Feedback + Instruct, and Feedback 5 blocks. Colored dashed lines correspond to the goal for that given dimension (purple for stroke-to-control, green for control-to-stroke). * denotes a between-subject difference (p < 0.05) and all error bars denote SEM

To gain additional insight into each group’s performance during Session 2, we analyzed the paretic hip and paretic knee angles over blocks of training (Fig. 9a, b). We observed that while stroke-to-control was not able to change their paretic knee flexion (F_2,16_ = 0.002, *p* = 0.998; Fig. 9c), they were able to increase their paretic hip flexion during swing (F_2,16_ = 5.03, *p* = 0.020; Fig. 9d). Post-hoc analysis revealed that stroke-to-control experienced greater paretic hip flexion in *Feedback 5* than *No Feedback 1* by approximately 4.75 degrees (*p* = 0.039; Fig. 9d). Control-to-stroke experienced both a change in paretic knee (F_2,16_ = 29.35, *p* < 0.001; *NoFB1* vs *FB5* p < 0.001; *NoFB1* vs *NoFB + instruct* p < 0.001; *NoFB + instruct* vs *FB5* p = 0.039; Fig. 9c) and paretic hip angles (F_2,16_ = 16.23, p < 0.001; *NoFB1* vs *FB5 p* = 0.260; *NoFB1* vs *NoFB + instruct p* = 0.001; *NoFB + instruct* vs *FB5 p* = 0.014; Fig. 9d) across blocks. Indeed, participants within the control-to-stroke group overshot the goal walking pattern when given solely verbal instruction and then were able to use the visual feedback to approach the goal in both the knee and hip (Fig. 9b). When comparing stroke-to-control to control-to stroke performance using mixed-design ANOVA with *joint* and *block* within-subject factors and a *group* between-subject factor, we observed a significant *block x group* interaction (F_2,34_ = 21.39, p < 0.001). Post-hoc analysis revealed that this interaction was driven by a significant difference between groups during *No Feedback 1* (mean pairwise difference = 13.7 degrees; *p* = 0.003) and *No Feedback + Instruct* (mean pairwise difference = 10.9; *p* = 0.013), which was reduced by *Feedback* 5 (mean pairwise difference = 1.7 degrees; *p* = 0.642).

### Biasing movement outside of baseline walking

**Fig. 10:**
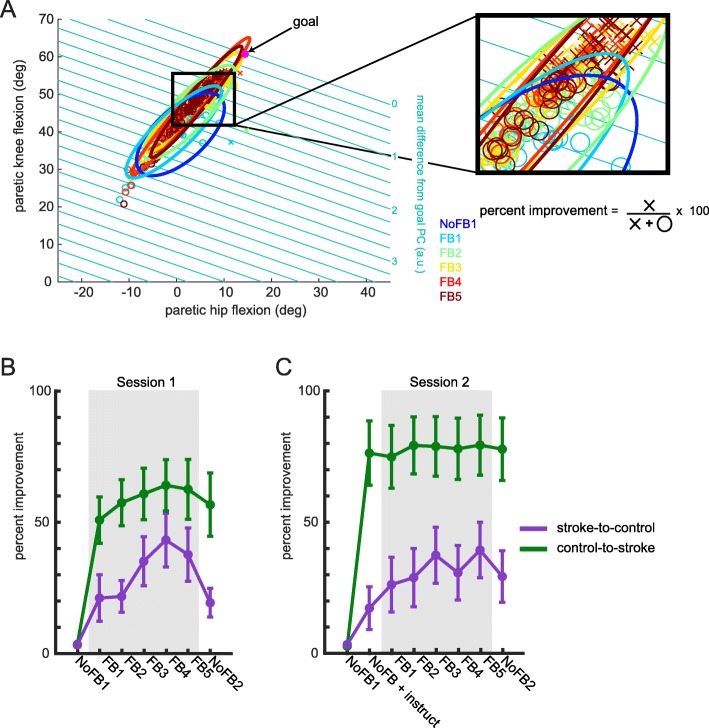
Percentage of steps taken outside of baseline walking pattern. (**a**) Calculation of percent improvement metric for sample stroke patient. Blue ellipse corresponds to a 95% confidence ellipse for baseline (No Feedback 1) paretic hip and knee flexion angles. Parallel teal lines denote combinations of paretic hip and knee flexion angles that represent the same deviation from the goal PC. Steps in subsequent training blocks are marked with an ‘x’ if outside of the baseline ellipse and closer to the goal PC. Steps in subsequent training blocks are marked with an ‘o’ if inside of the baseline ellipse or further from the goal PC. The inset displays steps around the border of the baseline ellipse to highlight this separation. (**b**) Percent improvement for stroke-to-control (purple) and control-to-stroke during Session 1 of training. (**c**) Percent improvement for stroke-to-control (purple) and control-to-stroke during Session 2 of training. Shaded regions in (**b**) and (**c**) correspond to blocks in which participants received visual feedback while walking. Error bars denote SEM

The purpose of the visual feedback was to bias participants away from their baseline walking pattern in the direction of the goal walking pattern. Thus, we wanted to quantify the percentage of steps in which participants were outside of their normal walking pattern during training using the *percentage improvement* metric. This metric describes the percentage of steps each participant experienced that was outside of their baseline variability for both paretic knee and hip flexion during mid-swing (defined as a 95% confidence ellipse from steps taken during *No Feedback 1*; blue ellipse, Fig. 10a) as well as demonstrating improved performance in PC space relative to the mean performance seen during *No Feedback 1*. Figure 10a displays this calculation over training for an example participant from the stroke-to-control group. Each symbol represents flexion values from the paretic hip (x axis) and knee (left y axis) during mid swing for each step. The teal lines represent the combination of paretic hip and knee flexion angles that would represent the same deviation from the goal PC (right y axis). Recall that the goal, noted here as a pink dot, can be represented in joint angle or PC space—this figure displays it in both ways. This was done to highlight the fact that the PC goal is simply a linear combination of the joint angles. Recall that the mean difference from goal PC was calculated with four input dimensions. Here, we focus on the paretic side joint angles. To condense this four dimensional calculation onto two dimensions for display, nonparetic flexion angles were set equal to the goal flexion angles. In this two-dimensional space, locking the nonparetic flexion angles would only result in a parallel shift in the teal performance lines and does not alter this analysis. The inset shows how we obtain our metric-- steps both outside of baseline variability *and* closer to the goal PC (i.e. improved performance) are marked by an ‘x’ and steps within baseline variability or farther from the goal PC (i.e. diminished performance) are marked by an ‘o’. The percent improvement is the proportion of improved performance steps over the total number of steps multiplied by 100.

Figure 10b displays group averages for *percent improvement* over the course of the Session 1 of training. Although both groups were able to increase their percentage improvement over blocks during Session 1, control-to-stroke was able to do so to a greater extent than stroke-to-control. Specifically, a mixed-design ANOVA revealed a significant *block* effect (F_1.37,21.96_ = 24.31, *p* < 0.001), *group* effect (F_1,16_ = 5.62, *p* = 0.031), and *block x group* interaction (F_1.37,21.96_ = 4.78, *p* = 0.030). Blocks included in this analysis were *No Feedback 1, Feedback 5,* and *No Feedback 2.* Post-hoc analysis revealed that stroke-to-control tended to reach a slightly lower amount of improvement by the end of training (*Feedback 5*, stroke-to-control: 38 ± 10, control-to-stroke: 62 ± 10; *p* = 0.056) and during *No Feedback 2* (stroke-to-control: 19 ± 6, control-to-stroke: 57 ± 10; *p* = 0.021).

Figure 10c displays group averages for *percent improvement* over the course of the Session 2 of training. A mixed-design ANOVA on ranked data revealed a significant *block* effect (F_3,51_ = 38.40, p < 0.001), *group* effect (F_1,17_ = 9.36, *p* = 0.007), and a *block x group* interaction (F_3,51_ = 9.14, p < 0.001). Blocks included in this analysis were *No Feedback 1, No Feedback + Instruct, Feedback 5,* and *No Feedback 2.*Of note, control-to-stroke was able to reach a higher level of improvement during the *No Feedback + instruct* (p < 0.001), *Feedback 5* (*p* = 0.044), and *No Feedback 2* (*p* = 0.028) than stroke-to-control. Still, stroke-to-control demonstrated an improved ability to explore beyond their baseline performance during *Feedback 5* (p < 0.001). Participants within stroke-to-control were not able to improve their percent improvement given solely verbal instruction (i.e. *No Feedback + instruct* vs *No Feedback 1*; *p* = 0.464). Thus, stroke-to-control was able to improve the quality of their steps beyond baseline only once they were given additional training with the visual feedback during Session 2.

## Discussion

These results demonstrate that principal component-based visual feedback is effective in simultaneously improving multiple features of walking in people with stroke within a single session of training. Moreover, the improvements were specific to each individual’s baseline walking deficits. Specifically, we found that people post-stroke were able to use the visual feedback to increase their paretic knee and hip flexion angles toward a more ‘healthy’ walking pattern. Additionally, a group of age-matched control participants were able to decrease their knee and hip flexion angle on a particular side to more closely match the goal kinematics of a hemiparetic walking pattern. This experiment investigated the use of the PC visual feedback when subjects were both unaware (Session 1) and explicitly aware (Session 2) of the goal walking pattern. Interestingly, while both groups were able to improve their performance when using purely exploration to find a more correct solution to the task, only the healthy controls were able to take advantage of the explicitly provided instruction to further improve performance. People post-stroke were unable to improve their performance once given information of the goal walking pattern.

### Patient-specific weighting of input dimensions

A key component of this novel visual feedback is its ability to individualize the performance feedback based on baseline deficits. Stroke often results in multiple deficits that negatively affect walking. Abnormal gait arises both from the direct consequences of neurological injury (e.g. decreased knee flexion due to paresis or spasticity) [2, 3] and from the compensations generated to mitigate those primary impairment (e.g. hip hiking or circumduction to compensate for decreased knee flexion during swing) [33]. This feedback algorithmically weights deficits in the inputted dimensions and is agnostic to whether they are due to impairment or compensation. Thus, the feedback guides participants to change multiple faulty features of their movement, whether from impairment or compensation, in order to achieve improved task performance.

Figure 4 highlights this feature of the visual feedback in which the dimensions that show greater deficits tend to be weighted higher when calculating feedback performance. For example, we can contrast the subject labeled with gold symbols who displays baseline deficits in all four input dimensions to the subject labeled with pink symbols who displays significant baseline deficits in only the paretic joint angles. The gold subject will be prompted to change aspects of walking within both the nonparetic and paretic step (all loadings above zero) while the pink subject will be prompted to change aspects of walking only within the paretic step in order to attain improved feedback performance. Note that the y-intercept of the best fit line is near zero, indicating that dimensions which do not display deficits will not be weighted using this feedback.

### Using PC feedback to alter multiple features of walking in people with stroke

We chose walking goals that necessitated each group to move in opposite directions so that we could demonstrate if the feedback led to differential changes in joint kinematics between groups. Indeed, we observed that the stroke-to-control group increased flexion angles while control-to-stroke group decreased flexion angles to more closely match their respective prescribed walking patterns.

The group of control participants experienced a larger improvement in performance than the group of people with stroke. This is not unexpected due to the presence of neurological pathology in our stroke-to-control group. Not only did they display baseline kinematic deficits due to either impairment or compensation, we also observed a difference in baseline cognitive function between groups. Stroke-to-control had lower MoCA scores (23 ± 2.9), compared to those of control-to-stroke (28.1 ± 1.0). Indeed, previous research has shown that cognitive decline impairs participants ability to improve in motor skill tasks [34–37]. Additionally, the ‘deficits’ imposed on the healthy control participants were due to our selected hemiparetic goal, which purposefully differed from healthy walking. Thus, healthy participants possessed a wider dynamic range to modify their walking pattern as they did not have the neurological constraints present in our stroke population. Although not explicitly tested, neurological damage following stroke places a limit on the capacity of some individuals to perform certain movements (e.g. achieve 60 degrees of paretic knee flexion while walking). Still, the intention of the ‘healthy’ walking goal was to bias the patients with stroke toward a healthier walking pattern within their capacity and not necessarily to achieve perfect performance. Therefore, decreased relative performance by the stroke-to-control group could have been due to motor impairment, cognitive impairment, or most likely a combination of both.

Interestingly, both groups displayed immediate retention of the modified walking pattern when the visual feedback was removed (Fig. 5e and f). In the *No Feedback* 2 block, participants were instructed to continue walking in a way that allowed them to achieve their best performance during the preceding feedback blocks. This retention of performance demonstrates that participants had some level of explicit awareness of how they were modifying their walking patterns to achieve improved performance. These results highlight the utility of visual biofeedback. It allows for participants to form an explicit connection between a desired outcome and their current motor output, beyond what is naturally available to them [38]. With this awareness, participants can self-correct aberrant features of gait. More work is needed to investigate retention over a longer period of time and the effect of repeated, prolonged exposure to this type of visual feedback.

### Adding movement-related instruction to PC feedback hinders performance in people with stroke

Control-to-stroke was extremely responsive to instruction (Fig. 8d, f). In fact, they overshot the goal following an instruction to walk with a hemiparetic gait (i.e. stiff knee and hip). Subsequently, they were then able to use the visual feedback to hone in on the goal until their performance was near-perfect (Fig. 8d). These results demonstrate that this novel PC feedback is effective in training an exact set of kinematics for a multi-dimensional walking pattern.

Notably, stroke-to-control was unable to alter performance beyond baseline during Session 2 (Fig. 8c). Once possible explanation for this finding is that the one participant who could not return for Session 2 (subject 8, Table 2) was responsible for driving the training effect observed during Session 1. To test if this was the case, we removed this subject from analysis for Session 1. Subsequently, we still observed a significant effect of training in Session 1(t_8_ = 3.88, *p* = 0.005), leading us to conclude that this subject’s absence from Session 2 is not what explains the disparity in performance between sessions.

We also speculated that the lack of improvement during Session 2 could be due to participants having already reached a performance ceiling during Session 1. Interestingly, baseline performance (i.e. *No Feedback 1*) during Session 2 did not differ from performance observed at the end of training (i.e. *Feedback 5*) during Session 1 (t_8_ = − 1.83, *p* = 0.104). This suggests that the lack of improvement during Session 2 may be due to a performance ceiling following Session 1.

When observing the kinematics, stroke-to-control was unable to increase their knee flexion beyond baseline levels. They were, however, able to increase their hip flexion by the end of training (Fig. 9d). Because the hip tended to be weighted less than the knee (Fig. 3d), these increases in flexion angle did not translate as much to performance in PC space as would an increase in knee flexion.

These results are surprising to us as conventional therapy heavily relies on instruction (e.g. bend knee more during swing, bring hip through faster, etc) to alter features of gait [39]. Verbal cues have been shown to increase muscle activity in paretic muscles in walking post-stroke [40]. In this same study, however, verbal cues did not have an effect on restoring symmetrical gait [40]. Therefore, it is possible that patients were co-contracting and making more effortful movements in response to instruction but not actually changing the movement itself.

Perhaps, the inclusion of joint-based instruction in Session 2 shifted the participants’ focus from external (i.e. improve performance using the visual feedback) to internal (i.e. try harder to bend my knee). In people post-stroke, verbal movement-related instructions has been shown to hinder motor performance compared to verbal task-related instruction [41]. Indeed, physical therapists tend to use more externally focused instruction during gait rehabilitation in stroke [39] as it has consistently shown to result in improved motor performance in healthy and pathological populations [42–44]. It appears that the shift to internal focus limited performance in this skilled walking task in people post-stroke. While future studies with a cross-over design are needed to confirm this hypothesis, it is worth considering where focus is directed when delivering therapy to patients post-stroke.

### People with stroke can use PC feedback to improve the quality of their steps during practice

Our analysis of percent improvement revealed that stroke-to-control was able to take a larger percentage of steps outside of their baseline walking during Session 1 and Session 2 (Fig. 10b, c). This suggests that patients are able to increase the quality of their practice (i.e. closer to healthy walking) while using the visual feedback. That is, approximately 30–40% of their steps are closer to a healthier walking pattern than they experienced when walking naturally. Moreover, people post-stroke appear to be able to maintain this higher quality of practice when the visual feedback is removed.

Previous work suggests that the greater repetition of high quality movements results in improved rehabilitation outcomes [45, 46]. From a motor learning perspective, a greater proportion of improved steps represents promise for engaging mechanisms (namely use-dependent plasticity (UDP) and reward-based learning) that could lead to long term changes in the motor repertoire. UDP refers to the process in which movement history biases subsequent movements toward the repeated movement [47] and has shown to increase with the learning of skilled motor task [48]. Reward-based learning occurs when different movements are associated with varying task outcomes [49]. Subsequent movements are biased toward the previously rewarded movement [50]. In the context of this task, the patients are not only repeating a ‘better’ walking pattern during training but they are also being rewarded for that improved pattern (i.e. via improved performance feedback). Both UDP [51] and reward-based learning [52] mechanisms have been shown to be present after stroke, thus providing promise for using visual feedback as a tool for long-term gait rehabilitation. Although we attribute improvements in this short-term study to improved performance, it is possible that with increased repetition and longer-term training that we can engage these learning mechanisms.

A limitation of this study is the focus on sagittal plane kinematics. While improved performance in this task was defined in terms of modified sagittal plane hip and knee flexions, it is likely that these were accompanied by changes in coronal and transverse plane kinematics, due to compensatory mechanisms. These compensations can either be beneficial or detrimental to the participant’s gait. Although this study limited the number of input dimensions to four kinematic variables, the number of inputs to be weighted can be expanded. This visual feedback system is flexible to the number of inputs and further implementation of this system can include kinematic variables in these additional planes of movement to further constrain compensatory movements. Thus, participants will only be able to improve in the task by modifying the primary deficit (such as paretic knee flexion) in the desired direction but also minimizing detrimental movement compensations (such as hip circumduction).

## Conclusions

Overall, these results suggest that our novel PC feedback is an effective tool for people post-stroke to correct multiple features of gait and attain repetition of higher quality steps during training. Moreover, the PC visual feedback described here allows for individualization of performance feedback. In this group of ten participants with chronic stroke, we observed a heterogeneous set of deficits. This algorithmic feedback accommodated this heterogeneity by weighting each walking feature accordingly to target patient-specific deficits. While long-term studies are needed to evaluate the use of PC feedback to more permanently alter gait, we believe these findings show promise for PC feedback as a tool for improving multiple patient-specific features of gait following stroke.

## Data Availability

Data available upon request.

## Abbreviations

ANOVA: Analysis of variance
FM-LE: Fugl-Meyer lower extremity
MoCA: Montreal Cognitive Assessment
PC: Principal component
PCA: Principal component analysis
UDP: Use-dependent plasticity
